# Knowledge, Attitudes, and Perceptions of Evidence‐Based Dentistry Among Final‐Year Dental Students: A Multinational Study Across 6 Countries and 8 Universities

**DOI:** 10.1111/eje.70136

**Published:** 2026-03-18

**Authors:** Danica Vidović Juras, Vlaho Brailo, Rosa María López‐Pintor, Božana Lončar Brzak, Jean‐Christophe Fricain, Adrien Naveau, Ali Abdullah Alqarni, José López López, Sonia Egido Moreno, Marcio Diniz Freitas, Jacobo Limeres Posse, Luis Monteiro, Luis M. Silva, Giovanni Lodi, Niccolò Lombardi, Elena Varoni, Raj Ariyaratnam, Rícheal Ni Riordain, Michael Escudier, Barbara Carey, Molly Harte, Rui Albuquerque

**Affiliations:** ^1^ Department of Oral Medicine University of Zagreb School of Dental Medicine Zagreb Croatia; ^2^ Department of Oral Diseases, Dental Clinic University Hospital Centre (UHC) Zagreb Zagreb Croatia; ^3^ Department of Dental Clinical Specialties, ORALMED Research Group, School of Dentistry Complutense University of Madrid Madrid Spain; ^4^ Department of Oral Medicine, Dental Faculty University of Bordeaux Bordeaux France; ^5^ Department of Oral & Maxillofacial Surgery and Diagnostic Sciences, Faculty of Dentistry Taif University Taif Saudi Arabia; ^6^ Department of Odontostomatology, Faculty of Medicine and Health Sciences (Dentistry) University of Barcelona Barcelona Spain; ^7^ Medical Surgical Area, Dental Hospital University Barcelona (HOUB) Barcelona Spain; ^8^ Oral Health and Masticatory System Group Bellvitge Institute of Biomedical Research (IDIBELL) Barcelona Spain; ^9^ School of Medicine and Dentistry University Santiago de Compostela Santiago de Compostela Spain; ^10^ UNIPRO—Oral Pathology and Rehabilitation Research Unit, University Institute of Health Sciences (IUCS), Cooperativa de Ensino Superior Politécnico e Universitário (CESPU) Gandra Portugal; ^11^ Dipartimento di Scienze Biomediche Chirurgiche e Odontoiatriche—Università Degli Studi di Milano Milan Italy; ^12^ University of Manchester Manchester UK; ^13^ Cork University Dental School and Hospital University College Cork Cork Ireland; ^14^ Faculty of Dentistry, Oral &Craniofacial Sciences King's College London London UK; ^15^ Department of Head and Neck Surgical Oncology Guy's and St Thomas' NHS Foundation Trust London UK; ^16^ Oral Medicine Guy's & St Thomas' NHS Foundation Trust (GSTT), Guy's Hospital London UK

**Keywords:** dental students, evidence‐based dentistry, knowledge, questionnaire

## Abstract

**Introduction:**

Evidence‐based dentistry (EBD) refers to the use of the best available evidence in combination with clinical expertise and patient values to guide clinical decision‐making. Whilst evidence‐based medicine (EBM) is widely adopted in medicine, EBD is much less frequently used in dentistry. Research into dental students' views towards EBD is limited. Understanding the views of this group is essential to increasing adoption of EBD among the dental profession.

**Aim:**

This study assessed final‐year undergraduate dentistry students' knowledge, perception, and attitudes towards EBD, and identified barriers to its routine use.

**Methods:**

A 3‐month cross‐sectional study was conducted among 276 final‐year dental students from eight European universities. Data were collected using an online questionnaire.

**Results:**

Students demonstrated good understanding of the principles of EBD and reported positive attitudes towards EBD, recognising its value in clinical decision‐making and professional development. Significant differences in knowledge around EBD were observed between countries. The most frequently reported barrier to the use of EBD was insufficient training (61.6%), followed by a lack of confidence in interpreting research findings (59.4%), and limited access to relevant journals (47.8%).

**Conclusion:**

Dental students acknowledge the importance of EBD, but their knowledge and skills in this area are limited. Insufficient training is the primary barrier, pointing to an urgent need for curriculum change. EBD should be integrated into undergraduate dental curricula, emphasising skills in literature search, critical appraisal, and applying evidence to clinical scenarios. Dedicated educational programmes are essential to prepare future dentists for evidence‐based care.

## 1 Introduction

The concept of evidence‐based medicine (EBM) first emerged in the 1980s, when a group of epidemiologists, led by Dr. David Sackett, started to teach clinicians how to critically appraise scientific literature in order to inform their clinical practice [1]. The term EBM was later coined by Guyatt [2] to describe the use of this critical appraisal of the literature, combined with clinical expertise and patient values, when making clinical decisions for individual patients.

The exclusive use of the best available evidence to inform clinical decisions is likely to lead to inappropriate or unrealistic therapeutic decisions for the patient in question [3]. Similarly, relying solely on clinical experience risks practice becoming outdated, potentially to the detriment of patients, and is an obstacle to development in practice [3]. The key, therefore, is to integrate the best available evidence with clinical experience, whilst taking into account the beliefs and values of the individual patient concerned, providing a more realistic and patient‐centred approach to care. EBM is used widely in medicine, providing a framework for clinicians to make shared decisions with patients that are both informed by clinical expertise and best available evidence [2]. Since its introduction, EBM has contributed significantly to the quality of healthcare research output, encouraging the development of standardised techniques for conducting clinical research [4]. EBM has been consistently associated with improved patient outcomes, care and safety [5].

In dentistry, the counterpart of EBM is evidence‐based dentistry (EBD). The introduction of EBD closely followed EBM, but despite this, EBD is less frequently implemented in routine dental practice [6]. Barriers suggested to the use of EBD in dental practice include insufficient time, lack of evidence relevant to dentistry, and simply a preference for clinical experience, either one's own or that of a colleague, over evidence to guide practice [6, 7]. In the UK, undergraduate dental students are taught EBD as part of their training, but the style, quality and integration of this teaching with real clinical practice, is highly variable [8]. This variability further impedes the uptake of EBD among dental practitioners.

In dentistry, the adoption of EBD is critical for advancing the quality of care and ensuring that dental treatments are based on scientific research and are effective, particularly as treatments become more advanced [6]. A broad base of high‐quality dental research is pivotal to the successful adoption of EBD into routine dental practice, but the implementation of EBD also requires dental professionals who understand the concepts of EBD, understand its benefits, and have the skills to practice it successfully [9]. Undergraduate dental curricula that emphasise the importance of EBD result in greater knowledge acquisition, improved attitudes, and increased confidence towards EBD [10].

In medicine, numerous studies have been carried out to assess the knowledge, attitudes and perception of medical students towards EBM [11, 12, 13, 14]. The data collected from these studies help to inform undergraduate medical curricula to best support understanding and uptake of EBM in medical students and newly qualified doctors. In dentistry, no Europe‐wide studies investigating dental student knowledge, perception and attitudes towards EBD exist. Without these studies, it is impossible to fully understand why EBD is less widely accepted than EBM. Assessing dental students' opinions on EBD is essential to promoting its integration into routine dental practice.

The aim of this study was to assess clinical exposure to EBD, as well as knowledge, attitudes, self‐perceived competence, and perceived barriers to EBD among final‐year dental students from eight European universities. By investigating the educational experiences and barriers faced by students, this study seeks to identify key areas where dental education can be improved to encourage better uptake and routine use of EBD in dental practice.

## 2 Material and Methods

A three‐month cross‐sectional study was carried out among final‐year undergraduate dental students at eight European universities: King's College London (England), CESPU University (Portugal), University of Zagreb (Croatia), University of Bordeaux (France), University of Milan (Italy), University of Santiago de Compostela (Spain), University of Barcelona (Spain) and Complutense University of Madrid (Spain). All participating institutions sought ethical approval for this study. All institutions provide dedicated EBD teaching as part of their dental undergraduate programmes, although this differs across settings. Details of the EBD curriculum at each participating university can be found in Table 1.

**TABLE 1:** Summary of EBD curriculum at participating universities.

Country	University	EBD curriculum
United Kingdom	King's College London	At King's College London, the Bachelor of Dental Surgery undergraduate degree includes a dedicated 15‐credit module titled “Evidence‐Based Dentistry and Research” in Year 1. The learning objectives of the module are to develop students' ability to critically evaluate scientific literature, apply research principles, and integrate evidence into clinical decision‐making
Portugal	CESPU University	At CESPU, evidence‐based dentistry is introduced in Year 1 through the compulsory unit Research Methodology and Statistics (Metodologia de Investigação e Estatística, 5 ECTS), which covers study designs, basic statistical analysis, and interpretation of research findings. It is then developed further in Year 2 within Preventive & Community Dentistry (Medicina Dentária Preventiva e Comunitária I 4 ECTS and II 4 ECTS), where the syllabus explicitly includes evidence‐based dentistry and critical appraisal of the scientific literature. These competencies are expected to be applied across subsequent clinical training, and the programme culminates in a Year 5 dissertation (5 ECTS), strengthening students' ability to work with and communicate scientific evidence
France	University of Bordeaux	At the University of Bordeaux, EBD foundations are introduced early in the undergraduate programme during the DFGSO preclinical cycle, specifically in DFGSO2 (Year 2), Semester 3, within the UE Compétences transversales (2 ECTS). This transversal unit includes “santé publique” (public health) and “recherche documentaire” (literature/documentary searching), and emphasises critical reading of scientific articles, providing the essential information‐retrieval skills that underpin the use of the best available scientific evidence from the start of dental training. These teachings, focusing on experimental research, clinical research, and epidemiology, are then progressively deepened, each year, throughout the entire curriculum
Croatia	University of Zagreb	At the University of Zagreb, EBD‐related competencies are mainly developed through coursework in statistics and population health. Quantitative skills are taught in “Statistics in dental medicine” (3rd year, 6th semester; 1 ECTS), supporting interpretation of research data and evidence in clinical reasoning. Population‐based reasoning that underpins evidence application is introduced earlier in “Social medicine and epidemiology” (1st year, 2nd semester; 3 ECTS)
Italy	University of Milan	At the University of Milan, Evidence‐Based Dentistry is taught as a dedicated compulsory course in Year 2 of the single‐cycle Dentistry programme, worth 5 ECTS. The published course aims are to provide students with the philosophical/scientific basis for an evidence‐based approach and the basic skills for evidence‐based clinical practice, with basic statistics highlighted among the prerequisites—linking statistical literacy directly to EBD competencies
Spain	University of Santiago de Compostela	At the University of Santiago de Compostela, EBD‐related training is introduced in Year 1 through “Bioestadística e Introducción a la Investigación” (6 ECTS), which provides the research and statistical foundations for reading and interpreting scientific evidence. It is then supported in Year 2 by “Salud Pública” (6 ECTS), and applied more directly in prevention/community settings in Year 3 through “Odontología Preventiva y Comunitaria” (6 ECTS), where students use epidemiology and population‐health approaches that underpin evidence‐based clinical decision‐making in prevention
Spain	University of Barcelona	At the University of Barcelona, EBD foundations are introduced in Year 1 through **“**Epidemiologia, Salut Pública i Bioestadística” (6 ECTS), which provides core skills in epidemiology/public health and biostatistics needed to interpret research evidence. In the second, third and fourth years, various modules, such as Introduction to Clinical Practice, Oral Medicine, and Dentistry for Medically Compromised Patients, among others, dedicate part of their curriculum to reinforcing Evidence‐Based Dentistry, through seminars and/or interactive case analyses. Finally, EBD is further strengthened in the later years through “Anàlisi i Preparació de Treballs Científics en Odontologia” (Year 5, 3 ECTS; optional), which focuses on analysing and preparing scientific work, supporting critical engagement with the literature alongside clinical training
Spain	Complutense University of Madrid	At the Complutense University of Madrid, EBD foundations are taught in Year 1 (Semester 1) through the core basic subject “Introducción a la Investigación y estadística” (6 ECTS). The course addresses the fundamental principles of scientific knowledge and the experimental research process, including the ethical framework governing biomedical research. Students are introduced to the essential structure of a scientific article, the principal biomedical databases, and leading scientific journals in dentistry. The course includes practical training in conducting database literature searches. It also develops a conceptual understanding of inferential statistics and its interpretation, enabling students to critically appraise and interpret key findings reported in scientific literature

Data were collected using a purpose‐designed online questionnaire, created based on similar studies carried out on medical students in different medical schools [11, 13, 14]. Following this a pilot study was carried out to identify and correct ambiguities, misunderstandings, or biases in the questions. A total of 12 students from final year in University College Cork were recruited. Each student was provided with a copy of the draft questionnaire and asked to comment on readability and ease of comprehension. All responses were collated and returned to the questionnaire design team for consideration and integration into the final draft questionnaire. The questionnaire included questions on student demographics, understanding of EBD, attitudes towards EBD, as well as perceived barriers to its application.

The participants were final‐year undergraduate dental students from the participating universities. Any students who had previously completed a medical degree were excluded.

Following pilot and finalisation, the questionnaire was uploaded to a survey software (Google Forms) and distributed to participants via email (Appendix S1). The questionnaire was anonymous, and no personal data apart from age, gender, country and university were collected. Before completing the questionnaire, participants were required to give their consent and confirm that they understood the objectives of the study. Participation in the study was on a voluntary basis. Participants were asked to rate 45 statements regarding knowledge, attitudes and experience of EBD using a scale ranging from “Strongly Disagree” to “Strongly Agree”. They were also given 8 potential barriers to the use of EBD and were asked whether they viewed each option as a barrier in their experience.

For all three domains of the questionnaire (i.e., knowledge, attitudes and practice), global score per student was calculated by summing numerical values of the responses. To do this, negatively worded items (i.e., statements reflecting lower knowledge, less professional attitudes and less practical experience) were reversely coded. Reverse coding assigned new numerical values to items worded in the opposite direction of the main construct, thereby ensuring that all items consistently reflect either a high or a low level of that construct. In addition, reverse coding helps to minimise acquiescence bias [15, 16].

Reverse coding was performed using the formula:

$$\begin{array}{c} \left(\text{maximum number of responses}+1\right)\\ \;-\text{numerical value of the response} \end{array}$$

In this way, on selected statements, value in the scale 5 was coded as 1 ((5 + 1) −5), value 4 was coded as 2 ((5 + 1) −4), value 3 was coded as 3 ((5 + 1) −3), value 2 was coded as 4 ((5 + 1) −2) and value 1 was coded as 5 ((5 + 1) −1). All statements that have been reversely coded are presented in Appendix S2. Furthermore, these statements are also marked with an ‘^a^’ in Tables 3, 4, 5.

Data were organised in a Microsoft Excel worksheet and stored in a shared Google Drive folder. Statistical analyses were performed using SPSS version 11. The Kolmogorov–Smirnov test was applied to assess the normality of data distribution. As the data were not normally distributed, non‐parametric methods were applied.

Nominal variables were expressed as proportions, whereas continuous variables were summarised as median values with interquartile ranges (IQR). Differences in nominal variables were examined using the chi‐square test, while differences in continuous variables were analysed with either the Kruskal–Wallis test with post hoc‐Bonferroni correction or Mann–Whitney test, as appropriate. Correlation between global knowledge, attitude and practice score was determined using Spearman rank correlation. Statistical significance was set at *p* < 0.05.

## 3 Results

A total of 276 students participated in the study, comprising 246 females (75%) and 83 males (25%). Total response rate was 35.9%, ranging from 11.8% to 83.9% depending on the institution (Table 2). There were no significant differences in gender distribution among countries. Median age of participants was 24 (range 22–25).

**TABLE 2:** Participant numbers and response rates by country.

Country	Participants (*n*, % response rate)	Female (*n*, %)	Male (*n*, %)	Difference in gender, *p*
United Kingdom	18 (11.8)	14 (77.8)	4 (22.2)	0.176
Portugal	59 (38.5)	48 (81.4)	11 (16.2)	
France	61 (61.7)	39 (63.9)	22 (32.4)	
Italy	47 (83.9)	35 (74.5)	12 (25.5)	
Spain	66 (30.4)	50 (75.8)	16 (23.5)	
Croatia	25 (27.8)	22 (88)	3 (12)	
Total	276 (35.9)	208 (75.4)	68 (24.6)	

### 3.1 Self‐Perceived Knowledge of EBD

Results on students perceived knowledge of EBD are presented in Table 3. The highest proportion of participants (174; 63%) strongly agreed with the statement “Evidence‐based dentistry involves critical appraisal of research findings to aid clinical decision making”. The highest proportion of participants (122; 44.2%) strongly disagreed with the statement “The power of a study is irrelevant when practicing evidence‐based dentistry”.

**TABLE 3:** Knowledge of evidence‐based dentistry.

Statement	Agreement (*n*, %)					Difference by gender	Difference by country (*p*)	Difference by country
	Strongly disagree 1	2	3	4	Strongly agree 5			Pairwise comparison
								Post hoc Bonferroni correction, *p*
Evidence‐based dentistry involves critical appraisal of research findings to aid clinical decision making	0	1 (0.4)	15 (5.4)	86 (31.2)	174 (63)	0.539	0.688	—
Evidence‐based dentistry focuses on the best available research without considering patient views^a^	37 (13.4)	79 (28.6)	60 (21.7)	60 (21.7)	40 (14.5)	0.415	< 0.0001*	FRA<POR; 0.008 FRA<ITA; < 0.0001 FRA<SPA; 0.019 FRA<CRO; < 0.0001
Evidence‐based dentistry focuses on the best available research without considering clinical expertise^a^	63 (22.8)	91 (33)	66 (23.9)	35 (12.7)	5 (7.6)	0.384	< 0.0001*	FRA<POR; 0.009 FRA<ITA; 0.001 FRA<SPA; 0.007 FRA<CRO; 0.003
Evidence‐based dentistry helps to promote self‐directed learning and continued professional development	0	9 (3.3)	27 (0.8)	101 (36.6)	139 (50.4)	0.089	0.537	—
Evidence‐based dentistry improves dentists' understanding of research methodology	2 (0.7)	5 (1.8)	40 (14.5)	103 (37.3)	126 (45.7)	0.072	0.001*	FRA<POR; 0.001
Evidence‐based dentistry can be used in situations where there is uncertainty about a clinical decision	1 (0.4)	4 (1.4)	21 (7.6)	107 (38.8)	143 (51.8)	0.200	0.131	—
Evidence‐based dentistry can allow for better standardisation of clinical care across settings	3 (1.1)	9 (3.3)	48 (17.4)	102 (37)	114 (41.3)	0.262	< 0.0001*	FRA<UK; 0.011 FRA<POR; < 0.0001 FRA<ITA; < 0.0001 FRA<SPA; < 0.0001 FRA<CRO; < 0.0001
Only dentists with an academic background (e.g., those with research experience or a PhD) can practice meaningful evidence‐based dentistry^a^	80 (29)	81 (29.3)	62 (22.5)	27 (9.8)	26 (9.4)	0.747	0.002*	FRA<POR; 0.007
The power of a study is irrelevant when practicing evidence‐based dentistry^a^	122 (44.2)	87 (31.5)	46 (16.7)	13 (4.7)	8 (2.9)	0.831	0.004*	Post hoc test did not find significant difference
Only meta‐analysis and systematic reviews should be used for evidence‐based dentistry^a^	52 (18.8)	84 (30.4)	78 (28.3)	41 (14.9)	21 (7.6)	0.802	< 0.0001*	ITA>POR; 0.003 ITA>FRA; 0.012 ITA>SPA; 0.023 CRO>POR; 007 CRO>FRA; 0.004 CRO>SPA; 0.007
Only Cochrane reviews should be used for evidence‐based dentistry^a^	86 (31.2)	68 (24.6)	81 (29.3)	27 (9.8)	14 (5.1)	0.908	< 0.0001*	POR>ITA; < 0.0001 POR>CRO; 0.002
When determining the efficacy of a particular treatment, a randomised controlled trial is superior to a case series	8 (2.9)	22 (8)	80 (29.0)	79 (28.6)	87 (31.5)	F > M 0.023*	< 0.0001*	SPA<ITA; 0.012
The PICO format (population, intervention, comparison, outcome) is a helpful way to formulate a clinical question for a literature search	1 (0.4)	6 (2.2)	59 (21.4)	111 (40.2)	99 (35.9)	0.332	< 0.0001*	FRA<POR; < 0.0001 FRA<ITA; < 0.0001 FRA<SPA; < 0.0001 FRA<CRO; 0.008
Audit is a type of research which could be used in evidence‐based dentistry^a^	12 (4.3)	59 (21.4)	116 (42)	64 (23.2)	25 (9.1)	0.913	< 0.0001*	SPA>FRA; 0.001 SPA>ITA; 0.001 SPA>CRO; 0.004
Case reports do not constitute high quality evidence and should never be used in evidence‐based dentistry^a^	40 (14.5)	87 (31.5)	95 (34.4)	37 (13.4)	17 (6.2)	0.063	0.333	—
Global knowledge score (median (IQR))	58 (53–62)					0.351	0.235	—

Abbreviations: CRO, Croatia; FRA, France; ITA, Italy; POR, Portugal; SPA, Spain; UK, United Kingdom.

^a^ The item was reversely coded for the calculation of general knowledge score.

* Significant difference (*p* < 0.05).

Significantly more female students agreed with the statement “When determining the efficacy of a particular treatment, a randomized controlled trial is superior to a case series” compared to male students (*p* = 0.023). No significant differences on other statements were found between male and female students. Significant differences among students from different countries was observed for nearly all statements. Distribution of student's responses to a particular question is presented in Figure 1A. Details in differences among countries for a particular statement are presented in Table 3.

**FIGURE 1:**
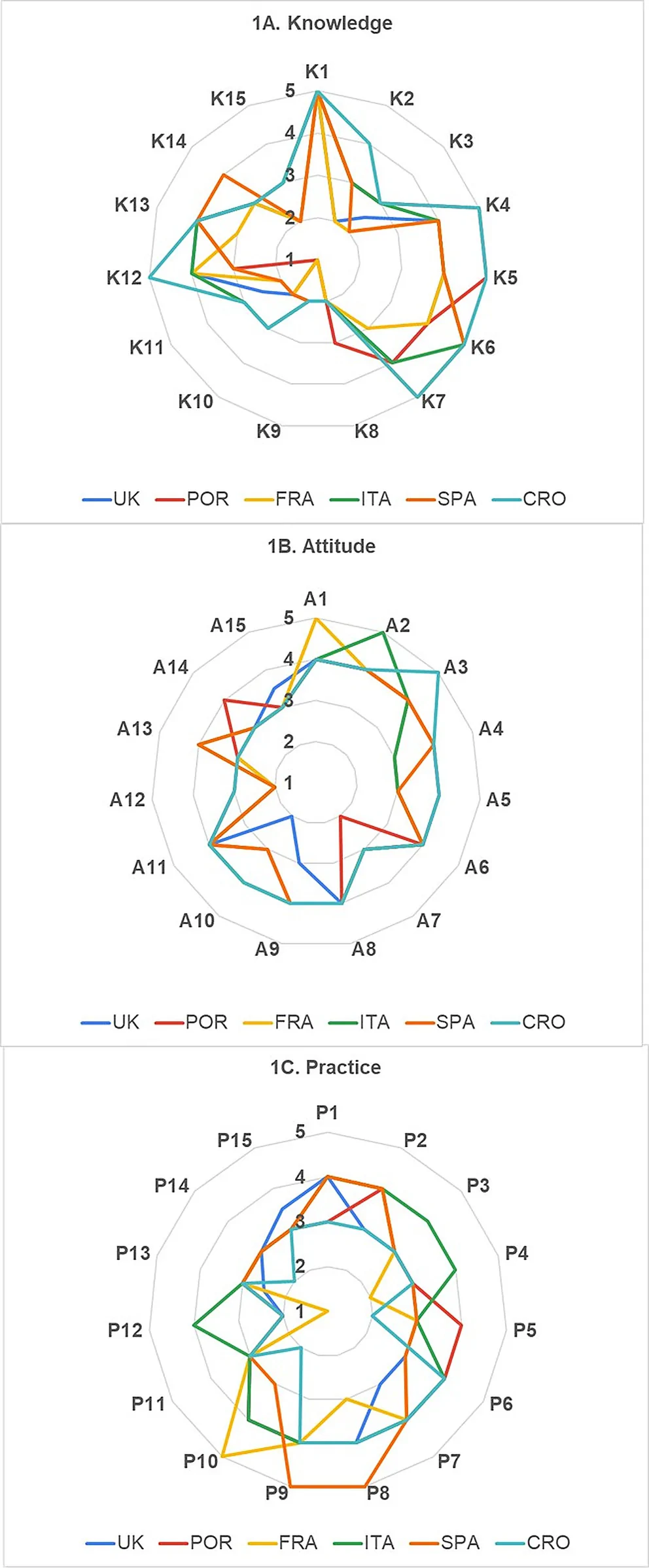
(A–C) Graphical representation of students' responses to each question by country. The rings represent median scores of each question on a 5—point Likert scale. CRO, Croatia; FRA, France; ITA, Italy; POR, Portugal; SPA, Spain; UK, United Kingdom.

Global knowledge score was 58 (53–62). No significant difference in global knowledge score was found between male and female students (*p* = 0.351) and among students from different countries (*p* = 0.235).

### 3.2 Attitudes Towards EBD

Results of students' attitudes towards EBD are presented in Table 4. The highest proportion of the participants (114; 41.3%) strongly agreed with the statement “I think that implementing evidence‐based practice will be of benefit to my continued professional development”. The highest proportion of the participants (57; 20.7%) strongly disagreed with the statement “I feel that evidence‐based dentistry is only relevant in specialist roles and is less applicable to general dental practice”.

**TABLE 4:** Attitudes towards evidence‐based dentistry.

Statement	Agreement N (%)					Difference by gender	Difference by country	Difference by country
	Strongly disagree 1	2	3	4	Strongly agree 5			Pairwise comparison
								Post hoc Bonferroni correction
I feel that there are benefits to making evidence‐based changes to my practice	1 (0.4)	4 (1.4)	39 (14.1)	130 (47.1)	102 (37)	0.063	0.121	POR<SPA; 0.001
I feel that applying evidence‐based dentistry to my practice increases the effectiveness of my work	2 (0.7)	3 (1.1)	39 (14.1)	136 (49.3)	96 (34.8)	0.714	0.338	—
I believe that research findings are very important in my day‐to‐day patient care	2 (0.7)	5 (1.8)	40 (14.5)	127 (46)	102 (37)	0.220	0.080	—
I feel years of clinical experience is more valuable than evidence‐based dentistry^a^	12 (4.3)	44 (15.9)	111 (40.2)	74 (26.8)	35 (12.7)	0.821	< 0.0001*	FRA<ITA; 0.027 FRA<SPA; 0.001 FRA<CRO; 0.001 POR>UK; 0.04 POR>FRA; 0.027
I feel more comfortable asking a senior colleague for an opinion on a clinical decision than relying on research papers^a^	9 (3.3)	37 (13.4)	109 (39.5)	85 (30.8)	36 (13)	0.126	0.006*	ITA<CRO; 0.001
I am keen to learn how to practice evidence‐based dentistry if given the opportunity	1 (0.4)	8 (2.9)	39 (14.1)	129 (46.7)	99 (35.9)	0.248	0.560	—
I feel that much of the available research is not applicable or relevant to my practice^a^	37 (13.4)	81 (29.3)	99 (35.9)	37 (13.4)	22 (8)	0.340	0.004*	CRO>POR; 0.031 CRO>FRA; 0.031
I think that implementing evidence‐based practice will be of benefit to my continued professional development	1 (0.4)	3 (1.1)	42 (15.2)	116 (42)	114 (41.3)	0.281	0.532	—
The requirement to practice evidence‐based dentistry places additional demands on me as a clinician^a^	5 (1.8)	19 (6.9)	85 (30.8)	109 (39.5)	58 (21)	0.808	< 0.0001*	UK<FRA; 0.001 UK<ITA; 0.015 UK<CRO; 0.005 FRA<SPA; 0.016
I think that practicing evidence‐based dentistry is time consuming^a^	9 (3.3)	33 (12)	90 (32.6)	83 (30.1)	61 (22.1)	0.618	< 0.0001*	UK<FRA; < 0.0001 UK<ITA; < 0.0001 UK<SPA; 0.028 UK<CRO; < 0.0001 POR<FRA; < 0.0001 POR<CRO; < 0.0001 SPA<ITA; 0.019
I feel that broader practice of evidence‐based dentistry would produce better dentists	1 (0.4)	4 (1.4)	59 (21.4)	136 (49.3)	76 (27.5)	0.235	0.487	—
I feel that evidence‐based dentistry is only relevant in specialist roles and is less applicable to general dental practice^a^	57 (20.7)	93 (33.7)	71 (25.7)	39 (14.1)	16 (5.8)	0.931	0.034*	Post hoc test did not find significant difference
I feel encouraged and supported by senior colleagues to practice evidence‐based dentistry	12 (4.3)	42 (15.2)	100 (36.2)	82 (29.7)	40 (14.5)	0.392	< 0.0001*	ITA>FRA; 0.012 ITA>SPA; 0.001 ITA>CRO; 0.021 SPA>FRA; 0.001 SPA>CRO; 0.004
I feel that patients would prefer treatment decisions based on clinical experience than treatment decisions based on high‐quality evidence^a^	13 (4.7)	53 (19.2)	102 (37)	74 (26.8)	34 (12.3)	0.539	0.173	—
I feel that evidence‐based dentistry can lead to high variability in treatments provided by different dentists^a^	19 (6.9)	49 (17.8)	90 (32.6)	80 (29)	38 (13.8)	0.850	0.260	—
Global attitude score (median (IQR))	51 (47–54)					0.351	0.003*	CRO<POR; 0.04 CRO<ITA; 0.021 CRO<SPA; 0.025

Abbreviations: CRO, Croatia; FRA, France; ITA, Italy; POR, Portugal; SPA, Spain; UK, United Kingdom.

^a^ The item was reversely coded for the calculation of general attitude score.

* Significant difference (*p* < 0.05).

No significant differences in agreement with any of the statements between male and female students was observed. However, significant differences in agreement among students from different countries was observed. Distribution of student's responses by country to a particular question is presented in Figure 1B. Details in differences between countries for a particular statement are presented in Table 4.

Global attitude score was 51 (47–54). No significant difference was found between male and female students in global attitude score (*p* = 0.351). Significant difference in global attitude score was found among students from different countries (*p* = 0.003). Students from Croatia had significantly lower global attitude score compared to students from Portugal (*p* = 0.04), Italy (*p* = 0.021) and Spain (*p* = 0.025).

### 3.3 Practical Experience of EBD

Results of students' reported practical experience of EBD are shown in Table 5. The highest proportion of students (119; 43.1%) strongly agreed with the statement “It is easy for me to access PubMed or other online bibliographic databases”. The highest proportion of the participants (157; 56.9%) strongly disagreed with the statement “I regularly participate in a journal club to keep up to date with new research and critical appraisal skills”.

**TABLE 5:** Self‐perceived practical experience of evidence‐based dentistry.

Statement	Agreement *N* (%)					Difference by gender	Difference by country	Difference by country
	Strongly disagree 1	2	3	4	Strongly agree 5			Pairwise comparison
								Post hoc Bonferroni correction
I have been taught about evidence‐based dentistry as part of my dental qualification	10 (3.6)	27 (9.8)	87 (31.5)	106 (38.4)	46 (16.7)	M > F; 0.005*	0.083	—
I have been taught how to conduct critical appraisal as part of my dental qualification	8 (2.9)	35 (12.7)	83 (30.1)	108 (39.1)	42 (15.2)	M > F; < 0.0001*	0.026*	—
I use evidence‐based dentistry to answer clinical questions in my usual practice	11 (4)	39 (14.1)	100 (36.2)	91 (33)	35 (12.7)	M > F; 0.021*	0.378	—
As a student, I do not currently use evidence‐ based dentistry, but I will use evidence‐based dentistry when I qualify as a dentist	33 (12.6)	43 (15.6)	100 (36.2)	73 (26.4)	27 (9.8)	0.077	< 0.0001*	FRA<POR; 0.001 FRA<ITA; < 0.0001 FRA<SPA; 0.014
I am confident in my ability to evaluate the quality of research papers	17 (6.2)	73 (26.4)	98 (35.5)	63 (22.8)	25 (9.1)	0.155	< 0.0001*	POR>FRA; 0.001 POR>SPA; 0.02 POR>CRO; 0.013
I know how to formulate a search question from a clinical problem to search for relevant research	6 (2.2)	58 (21)	88 (31.9)	88 (31.9)	36 (13)	0.049	0.002*	POR>FRA; 0.003
I know where to search for high‐quality up‐to‐date scientific research that can be used in evidence‐based dentistry	5 (1.8)	27 (9.8)	73 (26.4)	108 (39.1)	63 (22.8)	0.858	0.355	—
I regularly use PubMed or other online bibliographic databases to conduct literature searches	8 (2.9)	21 (7.6)	58 (21)	92 (33.3)	97 (35.1)	0.945	< 0.0001*	FRA<POR; 0.009 FRA<ITA; < 0.0001
It is easy for me to access PubMed or other online bibliographic databases	2 (0.7)	12 (4.3)	32 (11.6)	111 (40.2)	119 (43.1)	0.934	0.178	—
I have free access to a wide range of journal and publication subscriptions through my university	18 (6.5)	22 (8)	62 (22.5)	80 (29)	94 (34.1)	0.048	< 0.0001*	FRA>POR; < 0.0001 FRA>ITA; 0.004 FRA>SPA; < 0.0001 FRA>CRO; < 0.0001 CRO<UK; < 0.0001 CRO<ITA, 0.001
I find it hard to interpret research articles and their relevance to my practice^a^	17 (6.2)	60 (21.7)	110 (39.8)	71 (25.7)	18 (6.5)	0.869	0.076	—
I regularly participate in a journal club to keep up to date with new research and critical appraisal skills	157 (56.9)	43 (15.6)	33 (12)	26 (9.4)	17 (6.2)	0.450	< 0.0001*	ITA>POR; 0.015 ITA>FRA; < 0.0001 ITA>SPA; < 0.0001 ITA>CRO; < 0.0001 POR>FRA; 0.011 POR>SPA; 0.008
I do not have enough time to practice evidence‐based dentistry^a^	20 (7.2)	52 (18.8)	130 (47.1)	47 (17)	27 (9.8)	0.499	0.041*	UK>CRO; 0.042
Evidence‐based dentistry is well‐used and promoted among my peers	20 (7.2)	55 (19.9)	113 (40.9)	61 (22.1)	27 (9.8)	M < F; 0.012*	0.002*	ITA>CRO; 0.005
As a student, I feel it is more appropriate for me to ask a colleague for advice than conduct a literature search to answer a clinical question^a^	22 (8)	40 (14.6)	111 (40.5)	74 (27)	27 (9.9)	0.560	0.028*	Post hoc test did not find significant difference
Global practice score (median (IQR))	49 (45–53)					M > F; 0.015*	< 0.0001*	ITA>FRA; 0.011 ITA>CRO; < 0.0001 POR>CRO 0.015

Abbreviations: CRO, Croatia; FRA, France; ITA, Italy; POR, Portugal; SPA, Spain; UK, United Kingdom.

^a^ The item was reversely coded for the calculation of general practice score.

* Significant difference (*p* < 0.05).

Male students expressed significantly higher agreement with the following statements compared to female students: “I have been taught about evidence‐based dentistry as part of my dental qualification” (*p* = 0.005), “I have been taught how to conduct critical appraisal as part of my dental qualification” (*p* < 0.0001), and “I use evidence‐based dentistry to answer clinical questions in my usual practice” (*p* = 0.021). Male students expressed significantly lower agreement with the statement “Evidence‐based dentistry is well‐used and promoted amongst my peers” compared to female students (*p* = 0.012).

Significant differences were found among the students from different countries. Distribution of student's responses to a particular question is presented in Figure 1C. Details in differences between countries for a particular statement are presented in Table 5.

Global practical experience score was 49 (45–53). Male students had significantly higher global practical experience scores compared to female students (*p* = 0.015). Significant difference in global practical experience score was found among students from different countries (*p* < 0.0001). Students from Italy had significantly higher global practical experience score compared to students from France (*p* = 0.011) and Croatia (*p* < 0.0001). Additionally, students from Portugal had significantly higher global practical experience score compared to students from Croatia (*p* = 0.011).

### 3.4 Barriers to EBD

Table 6 presents perceived barriers to practicing EBD. Lack of training (i.e., “I do not feel I have had enough training on how to practice evidence‐based dentistry”) was perceived as a barrier by the highest proportion of participants (170; 61.6%). Lack of available relevant research (i.e., “I do not believe that the research available is relevant to my practice”) was perceived as the barrier by the lowest proportion of participants (91; 33%).

**TABLE 6:** Perceived barriers to practicing evidence‐based dentistry.

	Perceived as a barrier *N* (%)		Difference by gender	Difference by country	Difference by country
	Yes	No			Pairwise comparison
					Post hoc Bonferroni correction
I do not feel I have had enough training on how to practice evidence‐based dentistry	170 (61.6)	106 (38.4)	0.091	0.2	
I do not have access to the appropriate search databases	110 (39.9)	166 (60.1)	0.798	0.275	
I do not have access to the papers or journals needed, for example, subscription via institution	132 (47.8)	144 (52.2)	0.481	0.022*	Post hoc test did not find significant difference
I do not believe that the research available is relevant to my practice	91 (33)	185 (67)	0.673	0.157	
I do not have time to practice evidence‐based dentistry	145 (52.5)	131 (47.5)	0.186	0.687	
I do not feel confident in searching for high‐quality scientific literature	142 (51.4)	134 (48.6)	0.404	0.074	
I do not feel confident in interpreting the results of a research paper	164 (59.4)	112 (40.6)	F > M; < 0.0001*	0.230	
I do not have access to the resources needed to implement the best evidence‐based treatments	146 (52.9)	130 (47.1)	0.994	0.187	

* Significant difference (*p* < 0.05).

A significantly higher proportion of female students considered not feeling confident in interpreting the results of a research paper as a barrier to practicing EBD compared to male students (65.4% vs. 41.2; *p* < 0.0001). No significant differences in the perception of other barriers between males and females and among countries were found.

### 3.5 Correlation Between Global Knowledge, Attitude and Experience Scores

A significant moderate correlation was found between global knowledge and global attitude scores (*r* = 0.481; *p* < 0.0001). However, a null correlation was found between global knowledge and global practical experience scores (*r* = 0.015; *p* = 0.01). A weak correlation was found between global attitude and global practical experience scores (*r* = 0.283; *p* < 0.0001). Correlation coefficients are presented in Table 7.

**TABLE 7:** Correlation between global knowledge, attitude and practice scores.

	Global knowledge score	Global attitude score	Global practice score
Global knowledge score	—	0.481 *p* < 0.0001	0.015 *p* = 0.001
Global attitude score	0.481 *p* < 0.0001	—	0.283 *p* < 0.0001
Global practice score	0.015 *p* = 0.001	0.283 *p* < 0.0001	—

### 3.6 Impact of Perceived Barriers on Global Knowledge, Attitudes and Practical Experience Scores

Figure 2 presents the impact of perceived barriers on global knowledge, attitudes and practical experience scores. Students who perceived lack of training as a barrier to practicing EBD had significantly lower general attitude (*p* = 0.01) and practical experience scores (*p* < 0.0001) compared to students who did not perceive lack of training as a barrier to practicing EBD.

**FIGURE 2:**
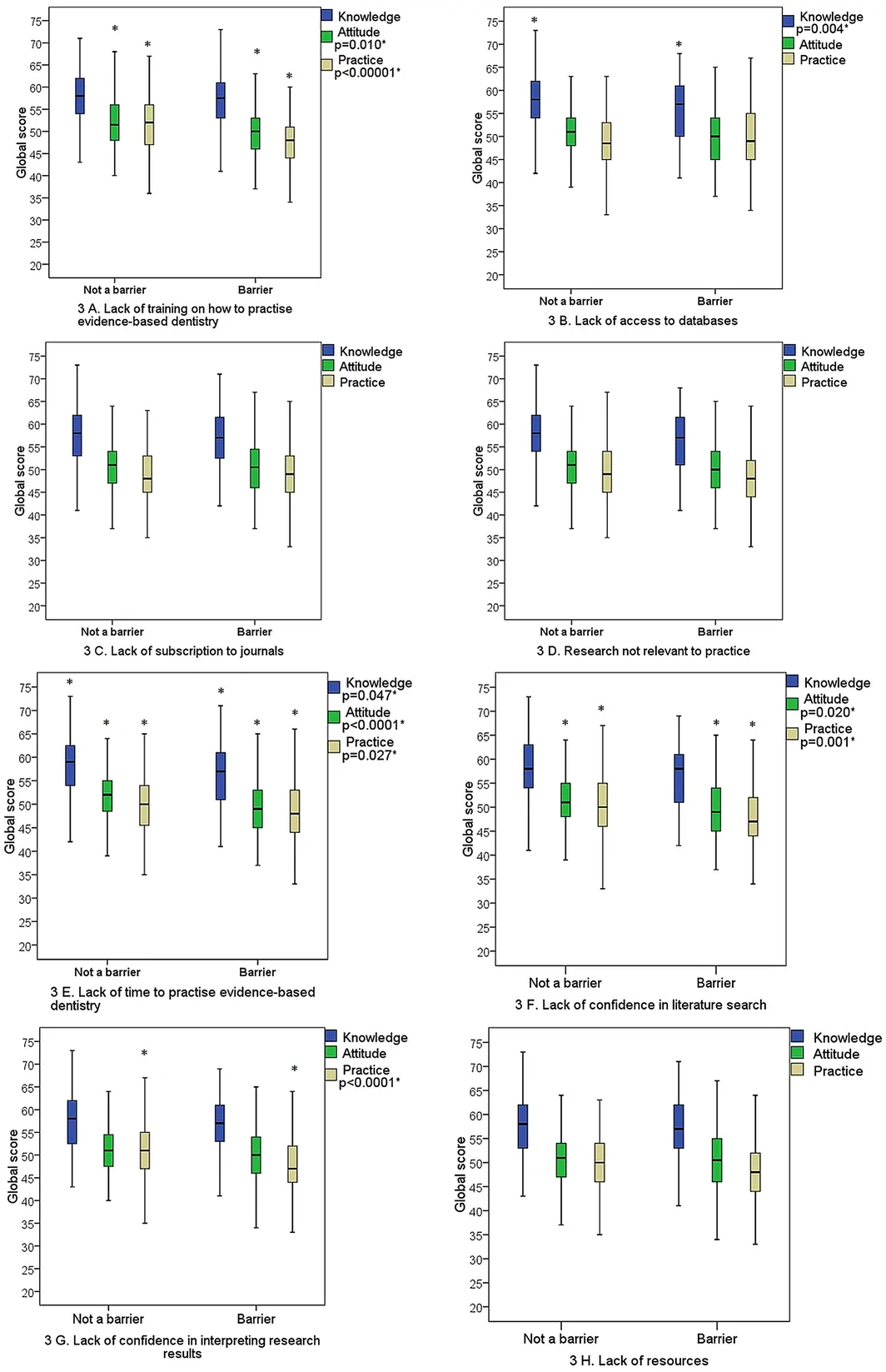
Graphical representation of differences in global knowledge, attitude and practical experience scores based on the perceived barriers for practicing evidence‐based dentistry. *; Significant.

Students who perceived the lack of access to databases as a barrier to practicing EBD had significantly lower global knowledge scores (*p* = 0.044) compared to students who did not perceive the lack of access to databases as a barrier to practicing EBD.

No significant difference in global knowledge, attitude and practical experience scores was observed between students who did and did not perceive lack of access to the papers or journals (*p* = 0.564, *p* = 0.524 and *p* = 0.717) as a barrier to practising EBD. No significant difference in global knowledge, attitude and practical experience scores was observed between students who did and did not perceive lack of relevance of research to their practice (*p* = 0.370, *p* = 0.402 and *p* = 0.151) as a barrier to practising EBD.

Students who perceived the lack of time as a barrier to practicing EBD had significantly lower global knowledge (*p* = 0.047), attitude (*p* < 0.0001) and practical experience (*p* = 0.027)scores compared to students who did not perceive the lack of time as a barrier to practicing EBD. Students who perceived the lack of confidence in literature search as a barrier to practicing EBD had significantly lower general attitude(*p* = 0.020) and practical experience (*p* = 0.001) scores compared to students who did not perceive the lack of confidence in literature search as a barrier to practicing EBD. Students who perceived the lack of confidence in interpreting the results of a research paper as a barrier to practicing EBD had significantly lower general practical experience scores (*p* < 0.0001) compared to students who did not lack of confidence in interpreting the results of a research paper as a barrier to practicing EBD.

No significant difference in global knowledge, attitude and practical experience scores was observed between students who did and did not perceive lack of resources (*p* = 0.655, *p* = 0.675 and *p* = 0.065) as a barrier to practicing EBD.

## 4 Discussion

EBD is a vital tool for providing effective, safe and high‐quality dental care to patients. In medicine, EBM has been associated with improved patient outcomes, care and safety [5]. Despite this, the level of knowledge, attitudes, and practical experience of EBD among dental students varies depending on educational programmes and available resources. Our results are in line with several international studies showing that dental students often have limited knowledge and practice about EBD but demonstrate a positive attitude towards its application [17, 18, 19, 20, 21]. In this study, students generally agree that implementing EBD would be beneficial for their continued professional development, but there persists a gap between this positive attitude towards EBD and the practical knowledge and skills required to implement it in practice. Previous studies have also shown positive attitudes of qualified dentists, with most dentists interested in learning about or adopting EBD‐related practices [22, 23, 24].

The majority of participants showed a sound understanding of the core principles of EBD. And most students correctly recognised that EBD involves critical appraisal of research evidence to inform clinical decisions and that randomised controlled trials represent a higher level of evidence compared to case series. These findings are consistent with previous studies among medical and dental students [10, 11, 12], suggesting that EBD concepts are being successfully introduced at the undergraduate level across Europe. However, substantial inter‐country differences were identified. Students from France consistently scored lower across several knowledge domains compared to peers from Portugal, Italy, and Spain. This may reflect differences in curricular emphasis, timing of teaching, and the extent to which EBD is integrated within clinical training (Table 1). For example, in France, EBD is taught early in the undergraduate programme, whereas in Portugal, EBD teaching is formally implemented throughout the undergraduate programme and culminates in a dissertation in the final year.

When asked about their attitudes towards EBD, overall attitudes towards EBD were positive, with most students agreeing that implementing EBD benefits professional development and enhances clinical effectiveness. This aligns with the growing recognition of EBD as a core component of lifelong learning and continuing professional development in dentistry [6]. The positive correlation between knowledge and attitude scores suggests that greater understanding of EBD principles fosters more favourable attitudes towards its practice, as seen in previous research among medical students [13]. Nevertheless, students from Croatia displayed significantly lower attitude scores than those from Portugal, Italy, and Spain. This may reflect lower institutional emphasis on EBD or fewer opportunities for experiential learning in critical appraisal. The perception that EBD adds time pressure or workload, reported by many students, also highlights the ongoing challenge of integrating EBD into an already busy clinical curriculum. This mirrors earlier findings by Sbaraini et al. [7], who identified “lack of time” as a key deterrent to EBD adoption in both student and professional settings.

Despite positive attitudes, results regarding self‐perceived practice of EBD were variable. Fewer than half of the students had received formal training in critical appraisal, and participation in journal clubs or structured literature reviews was rare. This gap between knowledge and practice has been widely observed in previous studies [6, 9]. Male students reported significantly higher exposure to EBD teaching and use of evidence to answer clinical questions, which could reflect either curricular variation or self‐perception biases in confidence. As our measures were self‐reported, we cannot disentangle true differences in teaching exposure from differences in confidence or response style. Consequently, gender‐related findings should therefore interpreted cautiously. Differences among countries were evident again: Italian and Portuguese students reported greater practical engagement with EBD than their French and Croatian counterparts. These findings underline the importance of curricular design particularly embedding EBD teaching within clinical settings and promoting active participation in research‐related activities. Students with higher practice scores may have benefited from integrated, competency‐based EBD training rather than isolated theoretical instruction.

A considerable number of students reported they frequently use PubMed or other databases (35.1%) and have access to dental journals where they could search for evidence (34.1%). This demonstrates an awareness of appropriate sources of evidence and willingness to use them, even if there is uncertainty about interpretation of the results of the scientific evidence. This is in line with other studies, reporting varying levels of database use such as 58.2% [18] and 39% (Fedorowicz et al. [25]).

When asked about perceived barriers to practicing EBD, lack of training was perceived as the barrier by the highest proportion of participants (170; 61.6%). Alongside this, they also reported lack of confidence in interpreting research findings and limited time. This correlates with the results from the existing literature, showing that in most universities, undergraduate curricula do not include critical appraisal or literature search skills essential to the adoption of EBD ([18]; Fedorowicz et al. [25]). Therefore, despite positive attitudes towards EBD, students face obstacles in implementing it. This highlights an urgent need for curricula update as regards EBD. Whilst we did not seek to compare the dental curricula across different universities in the present study, the significant differences in agreement among students from different countries regarding their knowledge, attitudes and clinical experience of EBD perhaps reflects shortcomings and variability in undergraduate curricula.

Changes in undergraduate dental curricula are key to improving knowledge and practical application of EBD. Marshall et al. [26] demonstrated successful outcomes from training students with the skills required for EBD. Various educational methods, including specialised courses and integrated curricula, have been documented as beneficial in improving students' perceptions and knowledge of EBD, especially in growing their practical skills of “finding” and “evaluating” evidence (Molena et al. [27]). Interestingly, access to journals or databases was not a major barrier for most students, perhaps reflecting improved digital access to research resources across European universities.

A moderate positive correlation was found between knowledge and attitude scores, indicating that improved understanding of EBD may cultivate more positive perceptions of its relevance and utility. However, the weak or null correlation between knowledge and practice underscores that theoretical instruction alone is insufficient to translate into applied competency. This “knowledge–practice gap” has been widely recognised in both dental and medical education [9, 12]. Active learning approaches including practical knowledge application, such as small‐group critical appraisal exercises, may bridge this gap by linking evidence interpretation to clinical decision‐making.

The main strength of the study lies in its international design, which allows for comparison of knowledge and attitudes towards EBD in different educational settings. The inclusion of final‐year students provides insights into students' level of knowledge, skill and experience immediately prior to independent practice. However, several limitations must be acknowledged. Firstly, the reliance on self‐reported data introduces the possibility of response bias, as students may over‐ or under‐estimate their knowledge and behaviours. Secondly, low response rates in some institutions may reduce the reliability of data collected. Thirdly, although we provide an overview of EBD curriculum from institution (Table 1), we did not conduct a formal curriculum audit to verify teaching content, assessment approaches, or the degree of clinical integration. Consequently, future research should systematically evaluate these curricular elements to better interpret differences observed between institutions.

Overall, the results of this study underscore a recurring theme in the literature showing that although students are aware of the importance of EBD and are increasingly using electronic sources of evidence, their ability to apply EBD effectively in clinical practice remains limited. Without structured, longitudinal training in literature search, critical appraisal, and interpretation of evidence, students may complete their dental studies with a positive attitude towards EBD but with insufficient practical skills to integrate it into clinical decision‐making. It is therefore necessary to review the undergraduate dental curricula in all settings studied to implement effective changes in this area.

## 5 Conclusion

This study highlights both strengths and limitations in the current state of EBD knowledge, attitudes, and practices among final‐year undergraduate dental students from eight European universities. Although dental students show good knowledge and a positive attitude towards EBD, their practical skills and confidence in applying EBD are often limited. The primary barriers identified were insufficient training, limited time, and lack of confidence in interpreting research. Strengthening EBD teaching through integrated, practice‐oriented educational strategies may educate the next generation of dentists who can effectively translate scientific evidence in general dental practice, thereby improving patient safety and outcomes.

## Funding

No funding was received for this study.

## Conflicts of Interest

The authors declare no conflicts of interest.

## Supporting information


**Data S1:** eje70136‐sup‐0001‐DataS1.docx.

## Data Availability

The data that support the findings of this study are available from the corresponding author upon reasonable request.
