# Supplementary material for: Knowledge, Attitudes, and Perceptions of Evidence‐Based Dentistry Among Final‐Year Dental Students: A Multinational Study Across 6 Countries and 8 Universities
Source: Eur J Dent Educ. 2026 Mar 18;30(4):1570–86. doi: 10.1111/eje.70136 (PMC13686660; doi:10.1111/eje.70136)
Supplement: Supplementary file 1 [file EJE-30-1570-s001.docx]

**Appendix 1**

Questionnaire distributed to participants

**Consent Statement**

I have read and understood the information provided and I agree to take part in this research project.

[ ]

**Demographic Data**

1. In which country do you study dentistry?

England

Ireland

Portugal

France

Italy

Spain

Croatia

2. What is your gender?

Male

Female

Non-binary

Other

Prefer not to say

3. What is your age?
4. Do you have any prior degree-level qualifications?

Yes, please specify

No

**Questionnaire**

Please indicate to what extent you agree / disagree with the following statements:

| **Knowledge of evidence-based dentistry** | Strongly Disagree | Disagree | Neutral | Agree | Strongly Agree |
| --- | --- | --- | --- | --- | --- |
| 1. Evidence-based dentistry involves critical appraisal of research findings to aid clinical decision making |  |  |  |  |  |
| 2. Evidence-based dentistry focuses on the best available research without considering patient views |  |  |  |  |  |
| 3. Evidence-based dentistry focuses on the best available research without considering clinical expertise |  |  |  |  |  |
| 4. Evidence-based dentistry helps to promote self-directed learning and continued professional development |  |  |  |  |  |
| 5. Evidence-based dentistry improves dentists’ understanding of research methodology |  |  |  |  |  |
| 6. Evidence-based dentistry can be used in situations where there is uncertainty about a clinical decision |  |  |  |  |  |
| 7. Evidence-based dentistry can allow for better standardisation of clinical care across settings |  |  |  |  |  |
| 8. Only dentists with an academic background (e.g. those with research experience or a PhD) can practice meaningful evidence-based dentistry |  |  |  |  |  |
| 9. The power of a study is irrelevant when practicing evidence-based dentistry |  |  |  |  |  |
| 10. Only meta-analysis and systematic reviews should be used for evidence-based dentistry |  |  |  |  |  |
| 11. Only Cochrane reviews should be used for evidence-based dentistry |  |  |  |  |  |
| 12. When determining the efficacy of a particular treatment, a randomised controlled trial is superior to a case series |  |  |  |  |  |
| 13. The PICO format (population, intervention, comparison, outcome) is a helpful way to formulate a clinical question for a literature search |  |  |  |  |  |
| 14. Audit is a type of research which could be used in evidence-based dentistry |  |  |  |  |  |
| 15. Case reports do not constitute high quality evidence and should never be used in evidence-based dentistry |  |  |  |  |  |
|  | | | | | |
| **Attitudes towards evidence-based dentistry** | Strongly Disagree | Disagree | Neutral | Agree | Strongly Agree |
| 1. I feel that there are benefits to making evidence-based changes to my practice |  |  |  |  |  |
| 2. I feel that applying evidence-based dentistry to my practice increases the effectiveness of my work |  |  |  |  |  |
| 3. I believe that research findings are very important in my day-to-day patient care |  |  |  |  |  |
| 4. I feel years of clinical experience is more valuable than evidence-based dentistry |  |  |  |  |  |
| 5. I feel more comfortable asking a senior colleague for an opinion on a clinical decision than relying on research papers |  |  |  |  |  |
| 6. I am keen to learn how to practice evidence-based dentistry if given the opportunity |  |  |  |  |  |
| 7. I feel that much of the available research is not applicable or relevant to my practice |  |  |  |  |  |
| 8. I think that implementing evidence-based practice will be of benefit to my continued professional development |  |  |  |  |  |
| 9. The requirement to practise evidence-based dentistry places additional demands on me as a clinician |  |  |  |  |  |
| 10. I think that practicing evidence-based dentistry is time consuming |  |  |  |  |  |
| 11. I feel that broader practice of evidence-based dentistry would produce better dentists |  |  |  |  |  |
| 12. I feel that evidence-based dentistry is only relevant in specialist roles and is less applicable to general dental practice |  |  |  |  |  |
| 13. I feel encouraged and supported by senior colleagues to practice evidence-based dentistry |  |  |  |  |  |
| 14. I feel that patients would prefer treatment decisions based on clinical experience than treatment decisions based on high-quality evidence |  |  |  |  |  |
| 15. I feel that evidence-based dentistry can lead to high variability in treatments provided by different dentists |  |  |  |  |  |
|  | | | | | |
| **Practice of evidence-based dentistry** | Strongly Disagree | Disagree | Neutral | Agree | Strongly Agree |
| 1. I have been taught about evidence-based dentistry as part of my dental qualification |  |  |  |  |  |
| 2. I have been taught how to conduct critical appraisal as part of my dental qualification |  |  |  |  |  |
| 3. I use evidence-based dentistry to answer clinical questions in my usual practice |  |  |  |  |  |
| 4. As a student, I do not currently use evidence-based dentistry, but I will use evidence-based dentistry when I qualify as a dentist |  |  |  |  |  |
| 5. I am confident in my ability to evaluate the quality of research papers |  |  |  |  |  |
| 6. I know how to formulate a search question from a clinical problem to search for relevant research |  |  |  |  |  |
| 7. I know where to search for high-quality up-to-date scientific research that can be used in evidence-based dentistry |  |  |  |  |  |
| 8. I regularly use PubMed or other online bibliographic databases to conduct literature searches |  |  |  |  |  |
| 9. It is easy for me to access PubMed or other online bibliographic databases |  |  |  |  |  |
| 10. I have free access to a wide range of journal and publication subscriptions through my university |  |  |  |  |  |
| 11. I find it hard to interpret research articles and their relevance to my practice |  |  |  |  |  |
| 12. I regularly participate in a journal club to keep up to date with new research and critical appraisal skills |  |  |  |  |  |
| 13. I do not have enough time to practice evidence-based dentistry |  |  |  |  |  |
| 14. Evidence-based dentistry is well-used and promoted amongst my peers |  |  |  |  |  |
| 15. As a student, I feel it is more appropriate for me to ask a colleague for advice than conduct a literature search to answer a clinical question |  |  |  |  |  |

**Barriers to evidence-based dentistry**

Please indicate which of the following you perceive to be a barrier to practising evidence-based dentistry.

| **Barrier** | **Perceived barrier** | **Not a barrier** |
| --- | --- | --- |
| I do not feel I have had enough training on how to practice evidence-based dentistry |  |  |
| I do not have access to the appropriate search databases |  |  |
| I do not have access to the papers or journals needed e.g. subscription via institution |  |  |
| I do not believe that the research available is relevant to my practice |  |  |
| I do not have time to practice evidence-based dentistry |  |  |
| I do not feel confident in searching for high-quality scientific literature |  |  |
| I do not feel confident in interpreting the results of a research paper |  |  |
| I do not have access to the resources needed to implement the best evidence-based treatments |  |  |

**Appendix 2**

List of statements that have been reversely coded for the calculation of the global knowledge, attitude and practice score

| **Statement number** | **Knowledge** |
| --- | --- |
| 2 | Evidence-based dentistry focuses on the best available research without considering patient views |
| 3 | Evidence-based dentistry focuses on the best available research without considering clinical expertise |
| 8 | Only dentists with an academic background (e.g. those with research experience or a PhD) can practice meaningful evidence-based dentistry |
| 9 | The power of a study is irrelevant when practicing evidence-based dentistry |
| 10 | Only meta-analysis and systematic reviews should be used for evidence-based dentistry |
| 11 | Only Cochrane reviews should be used for evidence-based dentistry |
| 14 | Audit is a type of research which could be used in evidence-based dentistry |
| 15 | Case reports do not constitute high quality evidence and should never be used in evidence-based dentistry |
|  | **Attitude** |
| 4 | I feel years of clinical experience is more valuable than evidence-based dentistry |
| 5 | I feel more comfortable asking a senior colleague for an opinion on a clinical decision than relying on research papers |
| 7 | I feel that much of the available research is not applicable or relevant to my practice |
| 9 | The requirement to practice evidence-based dentistry places additional demands on me as a clinician |
| 10 | I think that practicing evidence-based dentistry is time consuming |
| 12 | I feel that evidence-based dentistry is only relevant in specialist roles and is less applicable to general dental practice |
| 14 | I feel that patients would prefer treatment decisions based on clinical experience than treatment decisions based on high-quality evidence |
| 15 | I feel that evidence-based dentistry can lead to high variability in treatments provided by different dentists |
|  | **Practice** |
| 11 | I find it hard to interpret research articles and their relevance to my practice |
| 13 | I do not have enough time to practice evidence-based dentistry |
| 15 | As a student, I feel it is more appropriate for me to ask a colleague for advice than conduct a literature search to answer a clinical question |
